# Antibiotic consumption in surgical site infections treatment in Polish hospitals according to data from the program of active surveillance of hospital acquired infections

**DOI:** 10.1186/1753-6561-5-S6-P151

**Published:** 2011-06-29

**Authors:** A Różańska, J Wójkowska-Mach, M Bulanda, PB Heczko

**Affiliations:** 1 Chair of Microbiology, Jagellonian University Medical College, Kraków, Poland

## Introduction / objectives

The main scope of the HAIs control programs is quality of hospitalization improvement but the results of surveillance should also be used in benchmarking. The aim of this work was to assess and compare antibiotic usage in surgical site infections (SSI) treatment in Polish hospitals.

## Methods

Data used in this analysis were gathered in 13 hospitals participating in the program of active surveillance ofÂ HAIs in Poland between 2002/2006. In this time 96426 surgical procedures were done in hospitals which participated in the program. Altogether 2206 SSIs were detected in this population. Defined daily dose (DDD) was used for assessing antibiotic usage – per one SSI and per one operation.

## Results

Antibiotic usage in analyzed population varied in wide range: from 6,9 DDD/SSI to 45,5 DDD/SSI or from 0,1DDD to 1,6DDD per one operation. Similar differences were observed also when data according to type of procedure were analyzed; in abdominal surgery antibiotic usage varied from 5,1DDD to 122,6DDD per one SSI or from 0,1DDD to 4,4DDD per one operation of this type. In cardiac surgery the rate varied from 5,3DDD to 19,0DDD per one SSI or from 0,8DDD to 2,6DDD per one operation. In orthopedic surgery those rates reached the following values: from 1,7DDD to 136,4DDD per one SSI or from 0,1 to 21,4 DDD per one procedure. There were no significant differences observed in microbial etiological factors and their resistance.

## Conclusion

Presented data indicate huge discrepancies of antibiotic usage for SSI treatment in analyzed Polish hospitals. The results confirm the need of continuous developing and training in the infection control area, but also the need of developing reliable tools for benchmarking different aspect of infection control results.

## Disclosure of interest

None declared.

